# The Correlation of ELP4-PAX6 With Rolandic Spike Sources in Idiopathic Rolandic Epilepsy Syndromes

**DOI:** 10.3389/fneur.2021.643964

**Published:** 2021-04-09

**Authors:** Yiran Duan, Xuerong Leng, Chunyan Liu, Xiaohong Qi, Liping Zhang, Wenjun Tan, Xiating Zhang, Yuping Wang

**Affiliations:** ^1^Department of Neurology, Xuanwu Hospital, Capital Medical University, Beijing, China; ^2^Department of Pediatrics, Xuanwu Hospital Capital Medical University, Beijing, China; ^3^Key Laboratory of Intelligent Computing in Medical Image, Northeastern University, Ministry of Education, Shenyang, China; ^4^Beijing Key Laboratory of Neuromodulation, Capital Medical University, Beijing, China; ^5^Center of Epilepsy, Beijing Institute for Brain Disorders, Capital Medical University, Beijing, China

**Keywords:** idiopathic epilepsy syndromes, rolandic spikes, genetic, single nucleotide polymorphism, ELP4

## Abstract

**Objective:** To study the single nucleotide polymorphism rs662702 of ELP4-PAX6 in patients with idiopathic rolandic epilepsy syndromes (IRES) in China and explore the relationship between the distribution of rolandic spike sources and the single nucleotide polymorphism rs662702 in ELP4-PAX6.

**Methods:** First, clinical information was obtained from patients diagnosed with IRES. Next, the single nucleotide polymorphism rs662702 of ELP4 was analyzed by using the Sanger method. Resting-state magnetoencephalography data were collected from 17 patients. We analyzed the epileptic spike sources using the single equivalent current dipole (SECD) model and determined the spike distributions across the whole brain. Finally, Fisher's test was performed to assess the correlation between the single nucleotide polymorphism rs662702 of ELP4-PAX6 and rolandic spike sources.

**Results:** ELP4 rs662702 T alleles were found in 10.7% of IRES patients and occurred four times more frequently in these patients than in the healthy controls. TT homozygosity was found in one IRES patient (1.3%), while no TT homozygosity was found in the healthy control group. The IRES rolandic spike sources were unilateral in sixteen patients (94.1%) and were mainly located in the anterior central gyrus (58.8%). The spike source of patients without the ELP4 rs662702 T allele was correlated with the central region (*p* < 0.05). The rolandic spikes sources were significant correlated with the non-central gyrus (frontal and temporal lobes) in patients with the ELP4 rs662702 T allele (*p* < 0.05).

**Conclusion:** The rolandic spike sources of the IRES patients with the ELP4 rs662702 T allele were significantly associated with the non-central gyrus, including the frontal and temporal lobes. Our study confirmed for the first time *in vivo* that ELP4 rs662702 T allele overexpression is correlated with the rolandic spike distribution in patients with IRES and provides important insights into how genetic abnormalities can lead to brain dysfunction and into the precise targeting of abnormal discharge sources in the brain.

## Introduction

Idiopathic rolandic epilepsy syndromes (IRES) is the most common focal epilepsy syndrome occurring in children and is diagnosed based on age-dependent focal seizures, sharp-wave discharges, and normal brain MRI. IRES exhibit a phenotypic spectrum ranging from benign epilepsy with centrotemporal spikes (BECTS) (also called rolandic epilepsy) to atypical benign partial epilepsy (ABPE), Landau–Kleffner syndrome (LKS), and epileptic encephalopathy with continuous spikes and waves during slow-wave sleep (CSWS) (1, 2). BECTS, at the mild end of the spectrum, is represented by focal and secondarily generalized seizures and usually remits by adolescence. Electroencephalograms (EEGs) show centrotemporal spikes (CTSs) (mainly rolandic spikes) (3). ABPE, LKS, and CSWS, at the severe end of the spectrum, share the common EEG feature of rolandic spikes. They are characterized by more severe disorders with diverse seizure types and/or a highly pathological EEG. Patients with all of these types show language, cognitive, and behavioral deficits (4, 5).

While the genetic causes of IRES are largely undiscovered, family studies have proven that CTSs in IRES show an autosomal dominant pattern (6). Several studies have found GRIN2A mutations in the IRES spectrum, ranging in frequency from 4.9% in BECTS to 20% in CSWS, with mutations occurring significantly more frequently in the more severe phenotypes (5). Other rare sequence mutations have been identified in KCNQ2, KCNQ3, GABRG2, RBFOX1, and DEPDC5 (7–10). Copy number variations (CNVs) have been identified at the 16p11.2 locus in 1.53% of patients with BECTS and ABPE, but incomplete penetrance suggests that additional genetic and/or environmental factors exist (11). Linkage studies found CTS loci at 15q13.33, but evidence of CTSs in families with BECT was later reported at 11p13 in strong genome-wide linkage studies (12). Studies have confirmed that CTSs are associated with markers in ELP4, but no causative mutation has been found (13). However, recent family studies allowed the localization of allelic associations at the 11p13 locus for single nucleotide polymorphisms (SNPs) at ELP4-PAX6. PAX6, also located at 11p13, was finely mapped with ELP4, and the non-coding SNP rs662702 in PAX6 was linked with an increased risk of CTSs (OR = 12.29, *p* = 0.00026) (14). Thus, this site is potentially an interesting candidate site in IRES. *In vitro* analysis demonstrated that this SNP contributes to IRES by increasing PAX6 expression via the disruption of microRNA-328 binding.

CTSs are the primary EEG characteristic of IRES, and CTSs (rolandic spikes) propagate across the central area, indicating a more precise origin of epileptiform activity in the rolandic area. Thirty-nine EEGs from 27 patients with BECTS were examined for the pattern of spike propagation, and 32 foci in 20 patients showed a propagation pattern. Propagation across the central sulcus, from central to mid-temporal locations, is the typical pattern of rolandic spikes, suggesting that they originate from gyral or sulcal cortices on the central sulcus (15). The findings of EEG spike source dipoles in patients with BECTS and ABPE showed that the averaged spike source dipoles were oriented anteriorly in BECTS and posteriorly in ABPE (16). Compared with EEG, magnetoencephalography (MEG) is more sensitive for detecting spike sources using dipole modeling from sulcal and superficial epileptic foci and may be more beneficial for locating seizure foci than other methods (17). In 2003, equivalent current dipole (ECD) modeling was used to analyze the interictal spike sources in patients with BECTS, and the cortical generators for interictal spikes were determined to be localized in the precentral motor cortex, closer to the SII than to the SI cortex (18). In 2004, a spatiotemporal multiple signal classification (MUSIC) analysis of patients with BECTS suggested that the sources were spatiotemporally distributed and that the sources could propagate from the hand or finger area around the central sulcus down to the mouth or tongue area (18). A study from 2011 suggested that the spike source locations in the BECTS group were located significantly anterior, inferior, and lateral to those in the ABPE group. In the BECTS group, seizures tended to involve the mouth/tongue area rather than the hand/finger area in the ABPE group (19). Based on EEG and MEG source modeling analyses, most spike sources of IRES were localized in the central sulcus, and a few were located in other brain regions.

Although the present study analyzed the distributions and propagation characteristics of CTS sources and their associations with different clinical symptoms, no further research was conducted to determine the reasons for the differential distributions of the CTS origin, such as gene mutations, abnormal brain functional development, and environmental factors. We aimed to study the relationship between the distributions of CTS sources and gene variations in terms of rs662702 in ELP4-PAX6.

## Materials and Methods

### Patients and Healthy Controls

Case group: Seventy-five patients with clinically confirmed IRES were recruited from the outpatient department of Xuanwu Hospital from May 2017 to March 2019. Clinical identification of IRES was evaluated using the following inclusion criteria: (1) age of onset was between 3 and 13 years. (2) Focal seizures dominated by sensorimotor seizures and/or secondary to generalized tonic-clonic seizures. (3) EEG showed centrotemporal spike (CTS) discharge. (4) Physical examination showed no obvious neurological damage, and brain MRI showed no obvious structural abnormalities. (5) The diagnoses included BECTS, ABPE, LKS, and CSWS. The exclusion criteria for patients were as follows: (1) Patients with a history of neurological disease, head injury, or tumor. (2) Patients with severe internal medicine diseases, neuropsychiatric diseases, or abnormal growth and development. Control group: Seventy-five people who underwent routine physical examination were enrolled from Xuanwu Hospital from May 2015 to March 2019. The inclusion criteria were as follows: (1) healthy child or adult. (2) No previous history of febrile convulsion or epilepsy. (3) No consumption of any antiepileptic or antipsychotic drugs. (4) No history of neuropsychiatric diseases, trauma, tumor, or other neurological hereditary diseases. (5) No history of epilepsy among the individual or his relatives of no less than three generations. The exclusion criteria were as follows: (1) A history of neurological disease, head injury or tumor. (2) Severe internal medicine diseases, neuropsychiatric diseases, or abnormal growth and development.

### DNA Sequencing

Blood samples were collected from patients after they were clinically evaluated and diagnosed with IRES. Written informed consent was obtained from the legal guardians of the patients to share clinical, MRI, and EEG data and provide blood, as required by the institute's ethics committees. Patients with a genetic history of epilepsy (constituting at least one generation of the family) were enrolled together with their first-degree relatives (parents, siblings). From each participant, peripheral blood samples were collected, and genomic DNA was extracted using the Flexigene DNA Kit. The rs662702 in the 3′UTR of the ELP4-PAX6 gene was amplified by polymerase chain reaction (PCR) using intronic primers. The PCR products were purified by a QIAquick gel extraction kit, and commercial sequencing was conducted. The identified variants were sequenced at least twice in the forward and reverse directions. The nucleotide sequence was deposited under accession number BankIt2412891 NC_000016.10:c10182928-9753404 MW415422.

### MEG Recording and Analysis

Seventeen patients were subjected to MEG recordings with a 306-channel MEG system (Helsinki, ElektaNeuromag TRIUXTM) in a magnetically shielded room (MSR). Simultaneously, an EEG with 23 channels was recorded using the standard 10–20 international system. Both EEG and MEG signals were sampled at 1,000 Hz, bandpass filtered between 0.10 and 300 Hz, and recorded for 60 min under waking and sleeping conditions. To determine the position of the head relative to the MEG sensor during the acquisition process and to perform head movement correction later, five head position indicator (HPI) coils were placed on the scalp. A 3D Polhemeus digitizer was used to digitize the nasion, left and right preauricular points, HPI coils, EEG electrodes, and additional scalp points. These were used to transform the MEG coordinates on the MRI and to locate the head relative to the MEG system coordinates. MEG/EEG recordings were performed for 1 h, with 15 min time blocks being repeated four times, and the head position was measured before and after each block. During acquisition, head movement within the helmet <5 mm was acceptable, and 5 mm was the threshold for rejecting the MEG/EEG recordings. Visual inspection of the recorded raw data was performed to identify bad channels, and Elekta-Maxfilter software was used to preprocess data regarding head movement correction and artifact elimination. The current orientation of the main spike peak of the MEG was analyzed and related to negative polarity spikes that appeared in the simultaneous EEG. Identified spikes were collected and divided into three groups, unilateral spikes in the left hemisphere, unilateral spikes in the right hemisphere, and bilaterally synchronous spikes, which referred to the simultaneous occurrence of spikes on both sides; the left and right intervals of the main peaks were <50 ms (18). Single dipole modeling of MEG signals in a spherical head model was performed by using combined source modeling software (Neuromag, Helsinki). MEG source imaging (MSI) was used to project MEG sensors into the source space. MEG sources were localized by equivalent current dipoles (ECDs) at each time point for 100 ms before and after each spike. For statistical analysis, we used ECDs with an adequate goodness-of-fit (>85%) to analyze interictal spikes. Structural MRI data were obtained for each brain to construct an individual head model. Coregistration of source localizations on the structural MRI was performed using fiducial markers placed at the nasion and the right and left preauricular points to superimpose the magnetic source images of dipoles onto anatomic images. The MSI results were inspected, and the spike dipoles were classified according to their location and orientation. We measured each spike ECD position for each patient with reference to the median nerve somatosensory evoked fields (MN-SEFs), Broadmannin area, and location of the central sulcus. Then, we counted the ECD positions of the spikes of the patients.

### Data Analysis and Statistics

SPSS.19 software was used for the statistical analyses. The measurement data are presented as the mean values and variances to describe the centralized and discrete data trends. Fisher's test was used for hypothesis testing, and *p* < 0.05 was considered statistically significant.

## Results

### Clinical Data

Seventy-five patients with IRES who met the inclusion criteria were enrolled in this study. The main clinical data were as follows: The male-to-female ratio was 44:31, and the average age was 7.6 (range between 1 and 13, SD of 2.94). In total, 71 cases of BECTS (94.7%), three cases of ABPE (4%) (speech disorders and slow speech speed in one case, negative myoclonus in two cases), one case of CSWS (1.3%), 69 cases of unilateral onset (92%), 55 cases with oropharyngeal motor symptoms (73.3%), 19 cases with limb motor symptoms (25.3%), and 36 cases of secondary GTCS (48%) were included. There were 56 cases (74.7%) with no auras and 18 cases (24%) with auras (including two cases of facial numbness, one case of tongue numbness, two cases of hand numbness, one case of head tingling, two cases of headache, four cases of dizziness, one case of object enlargement and reduction in the brain, three cases of labored breathing, one case of abdominal pain, and one case of describing difficulty). Three patients (4%) complained of memory decline, including one patient with BECTS, one patient with ABPE, and one patient with CSWS. Four patients (5.3%) complained of attention decline, among which one patient was diagnosed with attention deficit hyperactivity disorder (ADHD) and one patient was diagnosed with ABPE. Thirteen patients (17.3%) had a history of febrile convulsions, including six with simple febrile convulsions and seven with complex febrile convulsions. Fourteen patients (18.7%) had a family history of epilepsy, and five patients (6.7%) had other systemic comorbidities but no brain structural abnormalities (including anemia, myocarditis, and patent foramen ovale).

### Gene Sequencing

Sanger sequencing and analysis of ELP4-PAX6 rs662702 in 75 patients with IRES and 75 healthy controls revealed the T/C allele and the corresponding genotypes of TT, CT, and CC. In the patient group, the genotype CC (80%) was the most common, followed by CT (18.7%) and TT (1.3%). The frequency of allele T was 10.7%, and the frequency of allele C was 89.3%. In healthy controls, the most common genotype was CC (94.7%), followed by CT (5.3%) and TT (0 cases, 0%); the allele T frequency was 2.7%, and the allele C frequency was 97.3%.

The genotypes (TT, CT, CC) and alleles (T/C) of ELP4 rs662702 in the patient and control groups were first subjected to the Hardy–Weinberg equilibrium test, and the difference was not statistically significant (*p* = 0.294, *p* > 0.05), indicating that the samples were in the genetic equilibrium state. Second, the Kruskal–Wallis rank-sum test was performed on the genotypes of the patient and control groups, and the results suggested statistically significant differences in the TT, CT, and CC genotypes between the patient group and the control group (*p* < 0.05). The allele distributions in the patient group and the control group were assessed by Fisher's exact test, with *p* = 0.005, suggesting that the T-allele frequencies in the patient group and the control group were significantly different, and the frequency of T alleles in the patient group was four times higher than that in the control group (Table 1).

**Table 1:** ELP4 RS662702 genotypes, allele distribution, and genetic analysis.

**ELP4 rs662702**	**Genotype**			**Allele**	
**Group**	**T/T**	**T/C**	**C/C**	**T**	**C**
Patients	1 (1.3%)	14 (18.7%)	60 (80%)	16 (10.7%)	134 (89.3%)
Healthy controls	0 (0%)	4 (5.3%)	71 (94.7%)	4 (2.7%)	146 (97.3%)
Genetic equilibrium	Hardy–Weinberg test: *p* = 0.294>0.05			Hardy–Weinberg test: *p* = 0.683>0.05	
Statistical difference	Kruskal–Wallis test: *p* = 0.007			Exact Fisher's test: *p* = 0.005	

### Clinical Phenotype Analysis of the ELP4 rs662702 Genotypes

Fourteen patients with the ELP4 rs662702 CT genotype were potentially pathogenic, among which 13 patients had BECTS and one patient had ABPE type 1, presenting with binocular left strabismus and mouth twitching to the left, followed by a generalized tonic-clonic seizure (GTCS) and negative myoclonus. One patient with the TT genotype was diagnosed with BECTS, presenting with a unilateral oropharyngeal twitch followed by a generalized tonic-clonic seizure (GTCS). The specific clinical features are summarized in Table 2.

**Table 2:** Abnormal genotypes and clinical phenotypes of ELP4 rs662702.

**Patient (diagnosis)**	**Onset age**	**Seizure frequency**	**Aura**	**Discharge side**	**Ocular symptoms**	**Oropharyngeal symptoms**	**Limb symptoms**	**Secondary GTCS**	**Others**	**Comorbidities**	**Family history**	**ELP4 rs662702**
ZSC (BECTS)	6	1 per month	–	L	Eyes blink	Salivate Sound in the throat	Bilateral limbs convulsions	+	–		+	TT
ZZY (BECTS)	9	0–1 per year	Right hand numb	L	Eyes look straight and blink	Salivate	Right limb convulsions	–	–	–	–	CT
YBR (BECTS)	9	2–3 Per months	Navel pain	L	Eyes right strabismus and blink	Salivate Mouth twitch to the right Sound in the throat	–	+	–	Complex febrile convulsion	–	CT
PYF (BECTS)	5	1 per month	–	L	Eyes right strabismus and blink	Salivate Mouth twitch to the right Sound in the throat	–	–	–	–	–	CT
FHT (BECTS)	5	4 per month	–	L	Eyes right strabismus and blink	Salivate Mouth twitch to the right Sound in the throat	–	+	–	–	–	CT
XXJ (BECTS)	9	1 per year	–	R	–	Salivate Lalopathy	–	–	Vomiting	–	–	CT
ZXW (BECTS)	11	2 per year	Headache	R	Eyes left strabismus and blink	Salivate Mouth twitch to the left Sound in the throat	Left limb convulsions	–	–	–	–	CT
YJ (BECTS)	10	2 per year	–	R	–	Salivate Mouth twitch to the right Sound in the throat	–	–	–	–	–	CT
ZJR (BECTS)	5	2–3 per year	–	R	Eyes right strabismus and blink	Mouth twitch to the right	Bilateral limbs convulsions	–	–	–	–	CT
HDY (BECTS)	13	2–3 per year	–	L	Eyes right strabismus	Salivate mouth twitch to the right Lalopathy	–	–	–	–	–	CT
SYZ (BECTS)	9	2 per month	–	R	Eyes left strabismus and blink	Salivate	–	–	Tinnitus	–	–	CT
QRX (BECTS)	7	2–3 per month	Headache	L	Eyes right strabismus and blink	Salivate mouth twitch to the right Lalopathy	Right limb convulsions	–	–	Simple febrile convulsion	–	CT
QZY (ABPE)	8	4 per year	–	R	Eyes left strabismus	Salivate mouth twitch to the left Lalopathy	Bilateral limbs convulsions	+	Negative myoclonus	–	–	CT
QYX (BECTS)	9	1 per month	–	L	–	Mouth twitch to the right Lalopathy	Bilateral limbs convulsions	–	–	–	–	CT
WXS (BECTS)	10	2 per month	–	R	Eyes left strabismus and blink	Salivate mouth twitch to the left Lalopathy	Left upper limb convulsions	–	–	Myocarditis	–	CT

### Magnetoencephalogram Dipole Distributions and Clinical Phenotypic Characteristics

Seventeen patients were selected to undergo a magnetoencephalography examination. The dipole distribution results are shown in Table 3. The MEG dipoles and spikes of each patient are shown in Table 4.

**Table 3:** Magnetoencephalogram dipole distribution statistics.

**Distribution location**			**Unilateral (case number)**	**Diagnosis**	**Bilateral (case number)**	**Diagnosis**
Noncentral region	Frontal lobe	Super frontal gyrus	1	BECTS	0	
		Middle frontal gyrus	1	BECTS	0	
		Inferior frontal gyrus	1	BECTS	0	
	Temporal lobe	Superior temporal gyrus	1	BECTS	0	
Central region (central sulcus, precentral gyrus, postcentral gyrus)	Precentral gyrus	Middle precentral gyrus	1	BECTS		
		Inferior precentral gyrus	8	7 BECTS 1 ABPE	1	CSWS
Others	Between middle frontal gyrus and inferior frontal gyrus		1	BECTS	0	
	Between middle frontal gyrus and precentral gyrus		1	BECTS	0	
	Between inferior postcentral gyrus and superior temporal gyrus		1	ABPE	0	

**Table 4:** MEG dipoles and spikes of each patient.

**Patient (diagnosis)**	**YBR (BECTS)**	**PYF (BECTS)**	**FHT (BECTS)**	**TQY (CSWS)**	**ZJZ (ABPE)**	**CWT (ABPE)**	**ZMQ (BECTS)**	**WYX (BECTS)**	**LJY (BECTS)**	**XZH (BECTS)**	**CDX (BECTS)**	**SRY (BECTS)**	**SBH (BECTS)**	**DHY (BECTS)**	**BYZ (BECTS)**	**GGH (BECTS)**	**LK (BECTS)**
ELP4 rs662702	CT	CT	CT	CC	CC	CC	CC	CC	CC	CC	CC	CC	CC	CC	CC	CC	CC
MEG dipoles distribution	Left inferior frontal gyrus	Left Middle frontal gyrus	Left Superior temporal gyrus	Bilateral anterior central gyrus	Left anterior central gyrus	Left posterior central gyrus	Right anterior central gyrus	Left anterior central gyrus	Left anterior central gyrus	Left anterior central gyrus	Left anterior central gyrus	Left superior frontal gyrus	Left anterior central gyrus	Left superior frontal gyrus	Right anterior central gyrus	Left anterior central gyrus	Left anterior central gyrus
Number of spikes passing the goodness-of-fit threshold	2	4	2	24	10	5	9	8	8	9	5	3	4	3	7	8	8

In one patient who was diagnosed with CSWS, the dipole was bilaterally distributed and located in the bilateral inferior precentral gyrus. The dipole distributions were unilateral in 16 patients, among which the dipole was distributed between the inferior postcentral gyrus and the superior temporal gyrus in one patient. The patient was diagnosed with ABPE and exhibited a corresponding aura of a tingling sensation throughout the whole head, and the clinical manifestations were oropharyngeal symptoms accompanied by negative myoclonus. In eight patients, the dipoles were distributed in the unilateral inferior precentral gyrus; seven were diagnosed with BECTS, with the corresponding clinical manifestations of oropharyngeal motor symptoms, and one was diagnosed with ABPE, with the clinical manifestations of oropharyngeal symptoms accompanied by an atypical absence, negative myoclonus, and language expression disorders. The dipoles of four patients were distributed in the frontal lobe, and these patients were diagnosed with BECTS, which was accompanied by oropharyngeal motor symptoms but no other specific symptoms or signs. One dipole was located in the superior temporal gyrus, and this patient was diagnosed with BECTS, with ocular and oropharyngeal motor symptoms as the main symptoms and no other specific clinical symptoms.

### Correlation Between the ELP4 rs662702 Genotype and CTS Source Distribution

**Figure 1:**
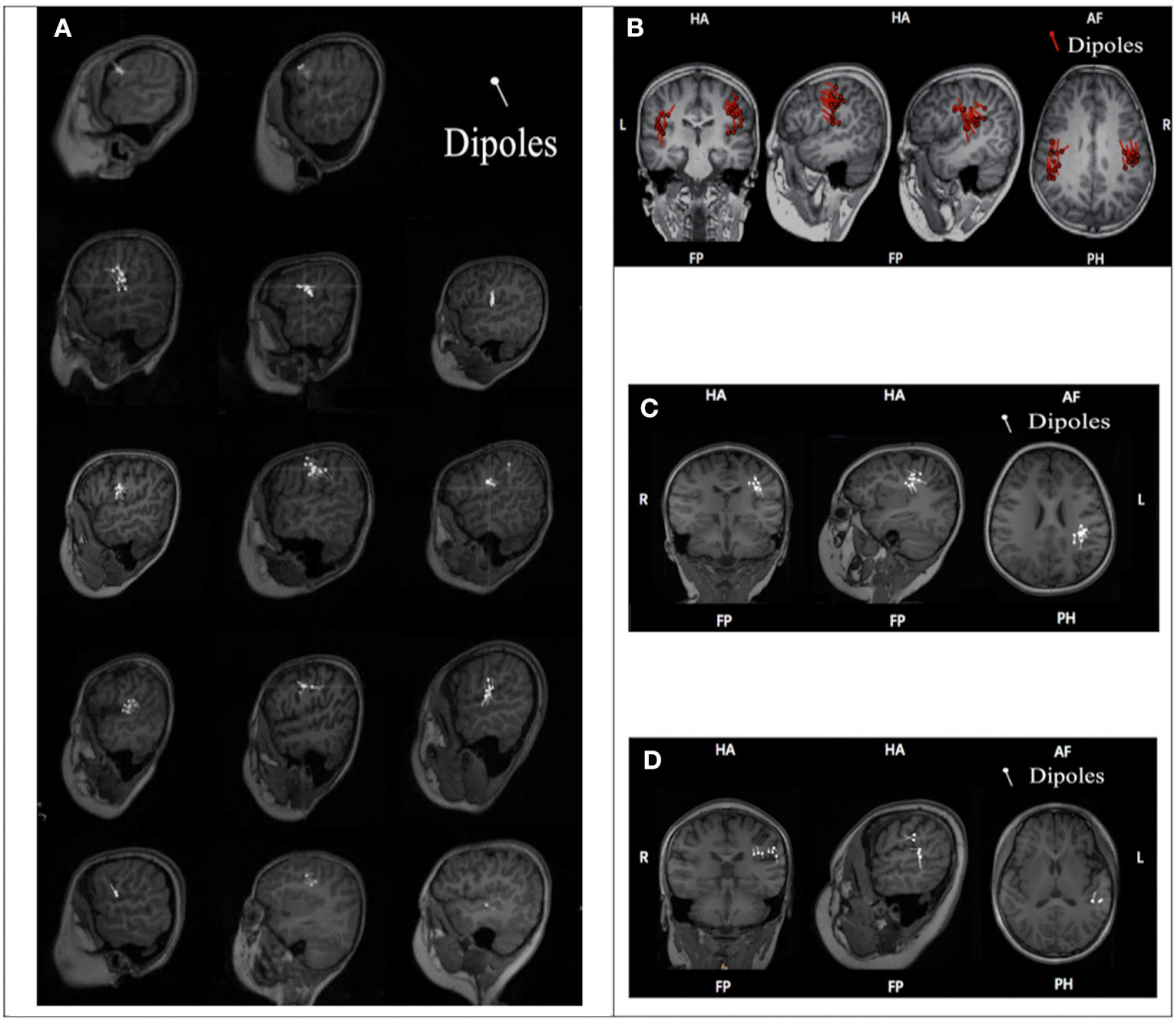
ELP4 rs662702 genotypes and CTS source distributions in 17 patients. **(A)** Genotype and distribution of CTS sources (dipoles) in 14 BECTS patients. Line 1: Two BECTS patients were ELP4rs662702 CC homozygous and their CTS sources were located in the frontal lobe. Lines 2–4: Nine BECTS patients were ELP4rs662702 CC homozygous and their CTS sources were mainly located in the central region. Line 5: Three BECTS patients were ELP4rs662702 CT homozygous and their CTS sources were located in the frontal gyrus, middle frontal gyrus, and superior temporal gyrus (from left to right). **(B)** The CSWS patient was ELP4rs662702 CC homozygous and his CTS sources were located in the bilateral inferior pre-central gyrus. **(C)** The ABPE patient was ELP4rs662702 CC homozygous and his CTS sources were located in the left inferior pre-central gyrus. **(D)** The ABPE patient was ELP4rs662702 CC homozygous and his CTS sources were located in the inferior post-central gyrus and superior temporal gyrus.

Among the 17 patients who underwent MEG examination, 3 BECTS patients had the ELP4 rs662702 CT genotype, and 14 patients (CSWS, ABPE, BECTS) had the ELP4 rs662702 CC genotype. The specific clinical classifications and distributions of the MEG CTS source locations, shown by dipoles, are shown in Figure 1.

We found that the CTS sources of three patients with the CT genotype were located in the unilateral non-central regions (in the frontal lobe in two patients and in the temporal lobe in one patient). On the other hand, the CTS sources of 10 patients with the CC genotype were located in the precentral gyrus. The sources were located in the inferior postcentral gyrus and the superior temporal gyrus in one patient with the CC genotype, located between the middle frontal gyrus and the precentral gyrus in one patient with the CC genotype, and located in the frontal lobe in two patients with the CC genotype. Therefore, Fisher's exact test was performed to study the correlation between the distribution of CTS sources in central or noncentral regions and patients with or without the T allele of ELP4 rs662702, and the results showed a statistically significant difference (p = 0.015) (Table 5).

**Table 5:** Discharge distribution and statistical analysis of the ELP4 rs662702 allele.

**Number of cases**	**Central region (central sulcus, precentral gyrus, postcentral gyrus)**	**Non-central region**
Patients with T allele (CT genome)	0	3
Patients without T allele (CC genome)	12	2
*P*-value	0.015	

## Discussion

On the spectrum of IRES, BECTS accounts for the majority of IRES, with a small percentage (1–7%) evolving to ABPE, LKS, or CSWS (20). In our study, BECTS accounted for 94.7% of IRES cases, ABPE accounted for 4%, and CSWS accounted for 1.3%, which is in accordance with the proportion of BECTS in IRES (87.2–94.6%) reported in a previous study (21–23). All patients enrolled in our study exhibited clinical and electrophysiological characteristics of IRES; however, we did not enroll patients with LKS due to the limited sample size.

The core phenotype of IRES is CTSs. The central temporal region includes the sensory and motor cortex around the central sulcus and has specific relationships with somatosensory and motor activities. Because the central temporal region mainly contains excitable pyramidal cells with a strong discharge capacity, it is an important region for epilepsy susceptibility. Therefore, it is crucial to accurately locate the sources of epilepsy susceptibility in the central temporal region to deeply understand the pathogenic mechanism of such diseases and provide guidance for accurate treatment.

To understand the complex genetic mechanisms of IRES, genome-wide association studies (GWASs) can help to find and locate complex disease-related gene SNPs and can be used to reveal complex genetic susceptibility and environmental factors. SNPs refer to the existence of two different bases at a specific nucleotide locus at the gene level, among which the least frequent occurs in more than 1% of the population. SNPs are mainly caused by the conversion or inversion of a single base and widely exist in the human genome. For the epilepsy susceptibility genes or SNP sites that have been discovered, the GWAS method still has the following limitations: it lacks accuracy for finding rare mutations in rare diseases, has the restriction of requiring associated functional sites and population heterogeneity, has a high false-positive rate, and has and poor repeatability. Therefore, further verification of the SNP loci of a certain gene by Sanger sequencing can accurately analyze the correlations between the SNP loci and different epileptic syndromes and even different traits and can accurately establish the correlation between a certain core phenotype and known genes. Our study focused on analyzing the relationship among CTSs, the core phenotype of IRES, and the SNP loci of the ELP4 gene.

In recent years, many studies have been conducted to explore the correlation between the ELP4 gene and BECTS, and few CTS-related disease-causing sites have been found (13, 24, 25). Numerous have finally focused on the T allele of SNP rs662702 in the 3′ untranslated region of ELP4-PAX6 (*p* = 0.00026), which is significantly correlated with CTSs traits (14). In our study, SNP analysis of ELP4-PAX6 rs662702 was performed for the first time in Chinese individuals, and there were significant differences in the T allele frequencies of the 75 IRES patients and 75 controls. The T-allele frequency of the IRES group was 10.7%, with a TT homozygous frequency of 1.3%, and the T-allele frequency of the healthy control group was 2.7%, with a TT homozygous frequency of 0. In a previous study, 152 individuals of European origin with CTSs were compared with 1,000 ethnically matched controls, and the T allele was present in 14% of the subjects in the CTSs group and in only 7.6% of those in the control group. TT homozygosity of the SNP rs662702 conferred an increased risk of CTSs (OR=12.29), which was found in 3.9% of the patients and in only 0.3% of the controls, suggesting that homozygosity at the T allele is a highly penetrable genotype. Five of the six patients with TT homozygosity for the SNP rs662702 had IRES, indicating that TT homozygosity may also lead to seizure susceptibility. Compared with previous studies, our study revealed similar T-allele frequencies and pathogenic trends among the patients with IRES. However, due to the small sample size and the absence of CTSs in patients with other diseases (such as autism and LKS), sampling errors may be present (13).

Elongation protein (ELP) plays a crucial role in the neural development of children by acetylating tubulin, regulating the growth of cortical projection neurons, and controlling the migration and differentiation of cortical neurons. The ELP4 protein is one of six elongator subunits, and a histone acetylase complex binds to the elongated form of RNA polymerase-2, which assists the polymerase and promotes DNA transcription. The ELP4 rs662702 T allele exerts pathogenic effects by reducing the binding affinity of microRNA-328, thus increasing PAX6 expression and interfering with its autoregulation. MicroRNA-328 degrades mRNA or reduces translation, which obstructs tubulin transport and further blocks the migration of projective cerebral cortex neurons. Experiments in embryonic mice suggested that the occurrence of the rs662702 T allele was related to CTSs and that overexpression of the rs662702 T allele led to cell-autonomous deficiency in the late proliferation of cortical progenitor cells. Specifically, overexpression caused cortical thickness and layering abnormalities in central and rostral regions (26), resulting in abnormal discharges in the corresponding cortical regions, and this mechanism was time-specific, mainly occurring during mid-childhood development. In addition, recent longitudinal MRI structural findings showed that CTS discharge was specifically distributed in the cerebral cortex (27). Compared with healthy people without CTSs, patients with CTSs exhibit frontal, temporal, and occipital cortical areas of reduced thickness, which supports that T-allele expression results in a specific and abnormal cortical thickness and further causes an epileptic discharge mechanism.

**Figure 2:**
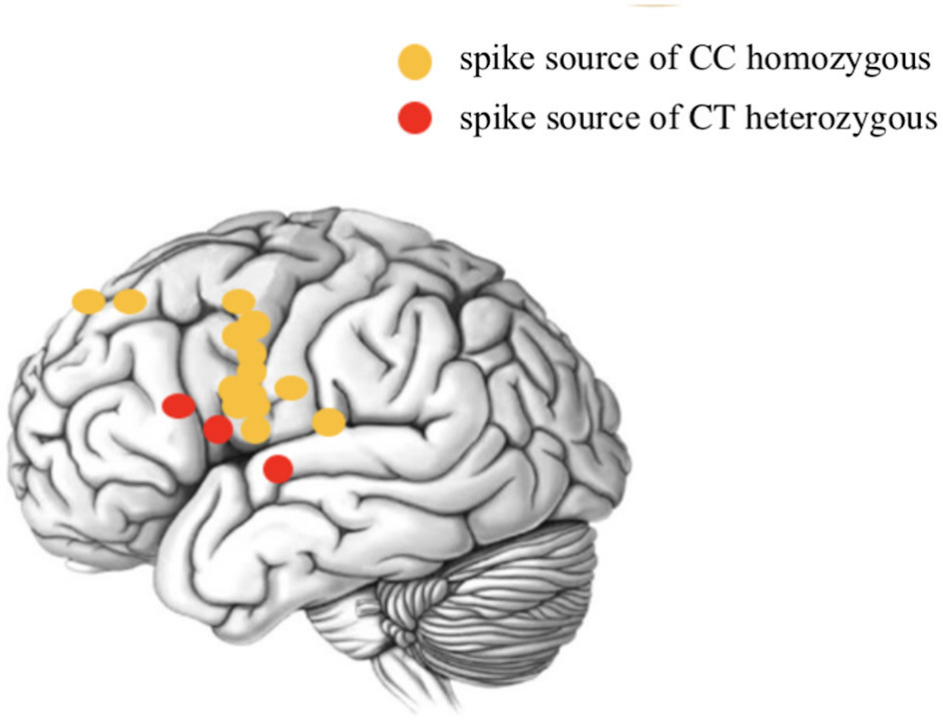
Schematic diagram of the CTS source distribution of ELP4 rs662702 in patients with different genotypes.

To further explore the relationship between the rs662702 T allele and the CTSs source, Sanger sequencing of ELP4 rs662702 and MEG dipole analysis of CTSs was performed in 17 patients, and the results suggested a significant correlation between patients with the ELP4 rs662702 T allele (three cases of CT heterozygosity) and patients without the T allele (14 cases of CC homozygosity) with the position distribution of the CTS origin (central and non-central regions). In patients with the T allele, the CTS source was mainly distributed in the non-central region, which was statistically significant (*p* = 0.004). Among the 3 CT heterozygotes, the CTS source was located in the frontal lobe in two patients and in the temporal lobe in one patient (Figure 2 below).

The results of our study are consistent with the specific distribution of CTS discharge in the brain area (frontal and temporal lobes) suggested by a previous study. Moreover, our study confirmed for the first time *in vivo* that ELP4 rs662702 T allele overexpression is associated with CTSs in patients with IRES and that CTS sources are differentially distributed in the brain. This lays the foundation for elucidating the epileptogenic mechanism of ELP4 gene variation in IRES and provides important insights into how genetic abnormalities can lead to brain dysfunction and then to the precise targeting of abnormal brain discharge sources. In addition, dysfunction or low expression of ELP can increase epileptic susceptibility and lead to language and cognitive impairment. Mutations in ELP4-related genes or loci can be further studied to explain the CTS discharge, speech, and cognitive decline characteristics of IRES. It should also be noted that the genetics of CTSs are not the same as those of BECTS. It has been reported that CTSs are autosomal dominant with age-dependent penetrance, and their incidence in normal children is 2–3% (28, 29). Its low penetrance indicates that BECTS is not only inherited via CTSs but also may be affected by multiple factors (1, 30, 31). CTS discharge has also been observed in fragile X syndrome, attention deficit hyperactivity disorder in children, developmental language disorders in children, etc., suggesting that brain injuries caused by various factors during childhood development can increase excitatory expression in the central temporal region (32).

This study has several limitations. First, it was conducted with a relatively limited number of patients; in particular, only 3/17 patients with CT polymorphisms and MEG source localization data were included, and more patients with CT polymorphisms will be enrolled in future studies to help to support the findings in our study. Second, dipole source localization was predicted by spherical head models, which lack proper representation of the head shape. Using the advanced boundary element method (BEM) or finite element method (FEM) could give more accurate calculations of scalp magnetic fields and produce relatively better results. Third, our study used MEG dipole modeling alone, and the combination of EEG and MEG should provide even more reliable source locations because EEG and MEG yield both confirmatory and complementary information.

## Conclusion

Our study confirms for the first time *in vivo* that ELP4 rs662702 T-allele overexpression is correlated with rolandic spike distribution in patients with IRES and provides important insights into how genetic abnormalities can lead to brain dysfunction and into the precise targeting of abnormal discharge sources in the brain.

## Data Availability Statement

The datasets presented in this study can be found in online repositories. The name of the repository and accession number can be found below: National Center for Biotechnology Information (NCBI) GenBank, https://www.ncbi.nlm.nih.gov/genbank/, MW415422.

## Ethics Statement

The studies involving human participants were reviewed and approved by Xuanwu Hospital Ethics Committee. Written informed consent to participate in this study was provided by the participants' legal guardian/next of kin. Written informed consent was obtained from the individual(s), and minor(s)' legal guardian/next of kin, for the publication of any potentially identifiable images or data included in this article.

## Author Contributions

YD collected and analyzed the clinical data, designed the study, and drafted the article. XL supervised the drafting and critically revised the article. CL, XQ, and LZ acquired the clinical data and critically revised the article. WT and XZ oversaw the data acquisition and performed the post-MRI processing. YW supervised the study, reviewed the literature, and revised the article. All authors contributed to the article and approved the submitted version.

## Conflict of Interest

The authors declare that the research was conducted in the absence of any commercial or financial relationships that could be construed as a potential conflict of interest.
